# Myositis-Myasthenia Overlap Syndrome After Immune Checkpoint Inhibitor Treatment: A Case Report

**DOI:** 10.7759/cureus.107600

**Published:** 2026-04-23

**Authors:** Christopher S Awad, Jack C Redick, Sydney Scheel, Neal Pohlman, Sorabh Khandelwal, Jason Bischof

**Affiliations:** 1 Emergency Medicine, The Ohio State University Wexner Medical Center, Columbus, USA; 2 Internal Medicine, The Ohio State University College of Medicine, Columbus, USA; 3 Anesthesiology, The Ohio State University Wexner Medical Center, Columbus, USA; 4 Internal Medicine, The Ohio State University Wexner Medical Center, Columbus, USA; 5 Emergency Medicine, The Ohio State University College of Medicine, Columbus, USA

**Keywords:** cardio-oncology, immune checkpoint inhibitors (icis), immune-mediated myasthenia gravis, immunotherapy-related myositis, oncologic emergencies

## Abstract

Immune checkpoint inhibitors (ICIs) are increasingly used in cancer therapy and are associated with immune-related adverse events (irAEs) that can mimic common emergency department (ED) presentations. These complications may resemble cardiopulmonary or neuromuscular disease, making early recognition challenging for emergency clinicians. We report the case of a 77-year-old man who presented to the ED with chest pressure and exertional dyspnea shortly after initiating immunotherapy. Initial evaluation focused on the treatment of a non-ST-elevation myocardial infarction. During his hospitalization, he developed progressive generalized weakness, ptosis, and worsening dyspnea with increasing oxygen requirements and reduced negative inspiratory force consistent with evolving respiratory compromise. He was ultimately diagnosed with probable ICI-associated myositis-myasthenia gravis overlap syndrome based on clinical findings despite negative serologic and electrodiagnostic testing. The patient was initially treated with high-dose intravenous methylprednisolone with transient improvement, but subsequently deteriorated after transition to oral corticosteroids. Empiric plasmapheresis was initiated, after which his respiratory status and neuromuscular strength gradually improved. He was discharged on a high-dose oral steroid taper with close neurology and oncology follow-up. This case highlights the diverse presentations of ICI-related irAEs and underscores the importance of obtaining a thorough oncology and immunotherapy history in patients presenting to the ED with unexplained cardiopulmonary or neuromuscular symptoms.

## Introduction

Immune checkpoint inhibitors (ICIs) are a form of immunotherapy that leverages the immune system to fight cancerous cells [1]. These humanized monoclonal proteins target immune checkpoint proteins and have been successful as primary or adjuvant therapies for many cancers [2]. However, treatment with ICIs can be complicated by a diverse array of immune-related adverse events (irAEs), including severe life-threatening presentations such as myositis, myocarditis, myasthenia gravis, pneumonitis, and hypophysitis, which may manifest after a single dose or be delayed by several months after the last dose [3,4].

Here, we present the case of a 77-year-old man who presented to the emergency department with signs concerning for decompensated heart failure and non-ST elevation myocardial infarction (NSTEMI) after a single treatment with nivolumab, who ultimately developed progressive hypoxia and weakness and was diagnosed with myositis-myasthenia gravis overlap syndrome.

## Case presentation

**Figure 1:**
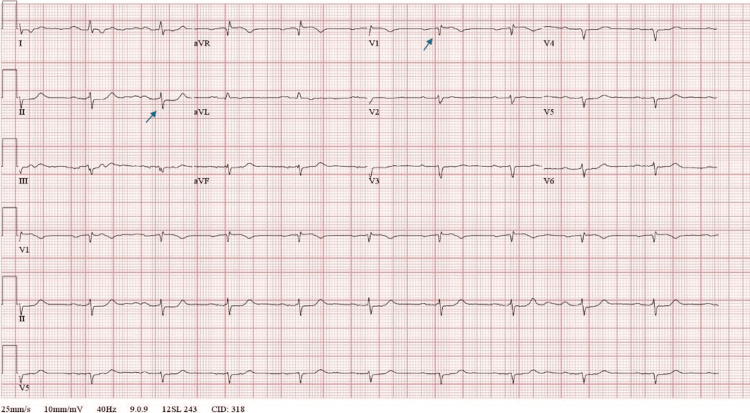
EKG on admission notable for ST depression in inferior leads (II, III, aVF) and anterior Q wave (lead V1).

A 77-year-old man with a past medical history significant for hypertension, type II diabetes mellitus, heart failure with preserved ejection fraction, chronic kidney disease, and a recent diagnosis of metastatic renal cell carcinoma (mRCC) presented to the emergency department (ED) as a transfer from an outside facility for NSTEMI. He initially presented with complaints of chest pain, back pain, increasing dyspnea on exertion, and orthopnea. Initial troponin was elevated to 2,167 ng/L with non-specific ST changes on EKG prompting transfer (Figure 1). Before transfer, he was given 325 mg of aspirin.

On arrival to the ED, his vital signs were a temperature of 37°C (98.6°F), blood pressure of 162/79 mmHg, heart rate of 62 beats per minute, respiratory rate of 18 breaths per minute, and oxygen saturation of 95% on room air. His physical examination was remarkable for a body mass index of 42.25 kg/m², bilateral lower extremity edema, and venous stasis changes, but was otherwise normal. His arrival EKG is demonstrated in Figure 1. His medication list included atorvastatin 10 mg, allopurinol 100 mg, vitamin D3, nifedipine 30 mg, prochlorperazine 10 mg, timolol maleate, cabozantinib 40 mg, hydralazine 100 mg twice daily, hydrocodone-acetaminophen 5-325 mg, and labetalol 200 mg twice daily.

Laboratory and diagnostic tests on presentation after transfer were significant for elevated high-sensitivity troponin (3,033, up-trending to 3,286 ng/L), B-type natriuretic peptide (118 pg/mL, reference range: 0-100 pg/mL), C-reactive protein (17.94 mg/L, reference range: <10 mg/L), and erythrocyte sedimentation rate (31 mm/hour, reference range: <20 mm/hour) (Table 1). Complete metabolic panel was unremarkable, and a complete blood count was consistent with a mild normocytic anemia. A chest X-ray was obtained and was only notable for mild cardiomegaly.

**Table 1:** Summary of clinically relevant tests and workup. Notably, the dates for the antibody panels were the date ordered/drawn, not the date of the result. The results took over a week to return. Peak values are in bold. Hs-Trop: high-sensitivity troponin; BNP: brain natriuretic peptide; ESR: erythrocyte sedimentation rate; CRP: C-reactive protein; CK: creatine kinase; LDH: lactate dehydrogenase; MG: myasthenia gravis; MuSK: muscle-specific receptor tyrosine kinase; EMG: electromyography

Date	Laboratory test	Value (units)	Reference range
Day 1	Hs-Trop	3,033 (ng/L)	<53
Day 1	BNP	118 (pg/mL)	<100
Day 1	ESR	31 (mm/hour)	<20
Day 1	CRP	17.94 (mg/L)	<10
Day 2	CK	7,362 (U/L)	30–220
Day 2	LDH	932 (U/L)	100–190
Day 2	Hs-Trop	3,935 (ng/L)	<53
Day 2	Hs-Trop	3,387 (ng/L)	<53
Day 3	CK	5,084 (U/L)	30–220
Day 7	CK	4,989 (U/L)	30–220
Day 8	NIF	-22 (cmH₂O)	>-30
Day 9	CK	3,750 (U/L)	30–220
Day 9	Aldolase	94.3 (U/L)	<7.7
Day 9	Paraneoplastic panel	Negative	-
Day 9	MG panel	0.00 (nmol/L)	<0.02
Day 9	Myositis panel	Negative	-
Day 9	MuSK antibody	0 (nmol/L)	0–0.02
Day 12	EMG and nerve conduction	No NMJ defect	-
Day 15	CK	599 (U/L)	30–220
Day 21	CK	193 (U/L)	30–220

His EKG showed sinus rhythm with a rate of 60 beats/minute (Figure 1). He was noted to have low-voltage, left-axis deviation, and Q-waves in the anterior precordial leads. The patient was started on a heparin drip and was admitted for further management of decompensated heart failure and NSTEMI, given rising troponin values and ongoing chest pain.

Upon admission to the hospital, the patient was treated like a presumed NSTEMI. Creatine kinase (CK) was elevated at 7,362 (U/L); hence, statin therapy was held as it was considered a potential etiology of the elevated CK (Table 1). Throughout the first week of his hospitalization, he had progression of his dyspnea and orthopnea, and maintained increasing oxygen requirement from a 2 L nasal cannula to 6 L despite diuresis. Further, he was unable to lie flat to tolerate left heart catheterization to definitively rule out acute coronary syndrome (ACS). After a week of workup and treatment, his clinical picture was not suggestive of cardiac disease as a driver of his pathology. More likely, his presentation was consistent with demand ischemia and a different, non-cardiac primary process; hence, pulmonology was consulted. At that time, the patient had progressed to a bilevel positive airway pressure (BiPAP) requirement for most of the day and all night. Pulmonology was not concerned for pulmonary disease as the CT chest did not reveal any pulmonary pathology. Rather, new examination findings of worsening proximal muscle weakness, right-sided ptosis, and right nasolabial fold asymmetry were suggestive of a neuromuscular cause of the patient’s dyspnea. On hospital day eight, neurology was consulted, and he was started on 40 mg of prednisone for three days due to concern for neuromuscular disease. He was found to have a negative inspiratory force (NIF) of around -20 cmH₂O. Electromyography (EMG) with repetitive nerve stimulation was not confirmatory for neuromuscular disease. Testing, including a myositis panel, muscle-specific kinase antibodies, a myasthenia gravis panel, and paraneoplastic antibodies, all came back unreactive. He showed some clinical improvement while on steroids and was able to tolerate left heart catheterization, which definitively ruled out ACS.

As the patient did not improve after the three days of oral prednisone, treatment was escalated to 1 mg IV methylprednisolone for five days (hospital days 11-15). His respiratory status improved while taking the high-dose methylprednisolone steroids; however, he began to deteriorate on his first day of the oral steroid taper (hospital day 16) and was started empirically on plasmapheresis. Following five plasmapheresis treatments over 10 days (hospital days 17-26), he was weaned off BiPAP to a 6 L nasal cannula. His NIF was variable following completion of plasmapheresis but eventually improved to -35 cmH₂O on hospital day 39. On hospital day 41, he was discharged to a rehabilitation facility in stable condition on a 2 L nasal cannula with an oral steroid taper and outpatient follow-up. At outpatient follow-up two months after discharge, he had completed therapy at the rehabilitation facility and had been breathing well on room air for over a month.

## Discussion

The patient’s ED presentation, with elevated troponins, EKG changes, and clinical signs and symptoms of volume overload, was initially most concerning for ACS and decompensated heart failure, which prompted admission. After coronary angiography demonstrated non-obstructive coronary disease, the patient’s chest pain and troponin elevation were attributed to demand ischemia secondary to uncontrolled hypertension and decompensated heart failure. However, over the course of his hospital stay, he developed worsening dyspnea and hypoxic respiratory failure and was ultimately diagnosed with ICI myositis-myasthenia overlap syndrome in the setting of a dose of nivolumab 24 days before his presentation at our ED. This presentation, in the context of malignancy and the recent administration of a programmed cell death protein 1 (PD-1) monoclonal antibody, should raise clinical suspicion for irAEs.

irAEs are known complications of ICIs. The drug he was given, nivolumab, works by targeting the PD1 receptor on T cells to prevent interaction with programmed death-ligand 1 (PD-L1) on cancerous cells [5]. A 2018 meta-analysis of irAEs demonstrated that the median time to irAE onset was 15 days following treatment initiation (range: 3-543 days), including 11 (52%) cases within 20 days, while the median time from symptom onset to death was 32 days (range: 3-355 days) [4]. This timeline of adverse events is particularly salient in the ED.

This presentation, consistent with a classic high-risk chest pain rule-out case, highlights the unique challenges of diagnosing irAEs in the ED setting as they commonly mimic the acute presentations of other disease processes. The ED team and referring facility failed to recognize his nivolumab administration, which should have led to the consideration of irAEs as a potential etiology for his shortness of breath.  During his admission, even though his CK remained elevated for two weeks (Table 1), the hospital team did not consider ICI-induced inflammatory complications of nivolumab until later in his hospital course (hospital day eight). His presentation on admission to the hospital team prompted concern for prolonged immobility and concurrent statin use, clouding the interpretation of elevated CK levels. This discussion further demonstrates the challenges of identifying ICI-related complications due to the possibility of multiple concurrent irAEs and the presence of overlap syndromes. Early inclusion of the treating oncologist’s input or consultation of an immunotherapy consultation service, if applicable, in the ED or early stages of the hospitalization may facilitate early identification of irAEs.

A rare and diagnostically challenging presentation of irAEs is a triad known as triple-M syndrome. This rare syndrome associated with a high mortality rate involves cardiac, musculoskeletal, and autoimmune dysfunction (myositis, myasthenia gravis, myocarditis) [3,6,7]. While this patient did not present to the ED with triple-M syndrome, he was eventually found to demonstrate symptoms consistent with two-thirds of this triad, myositis and myasthenia gravis. Although an endomyocardial biopsy or cardiac MRI was not performed to definitively rule out ICI-induced myocarditis due to clinical instability,  this potential irAE was thought to be less likely due to his troponin elevation normalizing over the first few days of admission. Further, this case exemplifies a common phenotype seen with the myasthenia-myositis overlap syndrome clinical picture, as patients are often seronegative without definitive EMG findings. This likely stems from the myositis itself having a predilection for oculo, bulbar, and respiratory muscles, giving the myasthenia picture in the overlap syndrome [8].

The patient’s EKG, a pivotal part of his clinical presentation, demonstrated low voltages and anterior Q-waves, and is challenging to interpret in the setting of his obese body habitus and his troponin elevation. EKG changes with concomitant chest pain and rising troponin levels may lead experienced providers to narrow their differential to ACS. With a thorough history and interrogation of medication changes, the ED physicians may have recognized the benefit of obtaining diagnostic inflammatory markers in the ED and broadening their differential to inflammatory causes of chest pain and shortness of breath that were not considered initially. This case report supports the need for maintaining a broad differential diagnosis when treating patients with cancer in the acute care setting to allow for early recognition of ICI-related complications and avoid the risk of cognitive biases such as search satisficing [9-11].

With a diagnosis of mRCC, or any cancer where immunotherapy is an efficacious treatment option, providers should be triggered to ask about medications and current treatments for these diseases. Pharmacologics such as ICIs are increasingly used for the treatment of cancers; up to 38.5% of patients with cancer were eligible for the use of an ICI in 2019 [12]. This is a meaningful population that is at risk for irAEs from ICI administration. As demonstrated in this case, the patient’s chest pain and shortness of breath were treated as a high-risk chest pain rule-out; if there was suspicion for irAEs from ICI medications, the mainstay of treatment in the ED would be steroids and plasmapheresis if the response to steroids is poor or the symptoms are severe [13]. The route and amount of steroids, and the need to supplement treatment with plasmapheresis, are generally based on the classification of the irAEs. Per the National Comprehensive Cancer Network guidelines, 500 mg to 1,000 mg of IV methylprednisolone is recommended for the initial treatment of severe neuromuscular irAE, with a steroid taper based on improvement, plus plasmapheresis or intravenous immunoglobulin if needed [14]. In the setting of a suspected adverse event to PD1 inhibitors such as nivolumab, medications such as tocilizumab may be administered in the management of steroid-refractory irAEs [13].

Additionally, the 2022 Model of the Clinical Practice of Emergency Medicine recently added immunotherapy complications to the scope of medical knowledge, patient care, and procedural skills that emergency medicine physicians need to recognize, and emergency medicine physicians managing patients who have been treated with ICIs should be aware that irAEs are common in this patient population [15].

As it can be challenging to disambiguate the etiologies of clinical syndromes in the emergency setting, this case highlights the need for emergency medicine physicians to anticipate oncologic emergencies within their scope of practice, recognize the widening scope of irAE mimics for common presentations in the ED, develop thorough histories from patients, and interrogate recent medication changes that may not be listed in patients’ charts [16].

## Conclusions

Presentations of complications related to immunotherapies are increasingly common in the ED and have recently been recognized as a key part of the scope of emergency medicine practice. We present a case of chest pain, exertional dyspnea, and elevated CK in the setting of starting ICI-therapy, initially treated as decompensated heart failure and NSTEMI that went on to be diagnosed with ICI-induced myositis and myasthenia gravis overlap syndrome. This case adds to the calls for early consideration, recognition, and treatment among emergency medicine practitioners of ICI complications, as this may reduce the morbidity and mortality associated with the disease.
